# A Novel Method for Precision Measurement and Result Optimization of Detuning Angle for KDP Crystals

**DOI:** 10.3390/s24020624

**Published:** 2024-01-18

**Authors:** Honghong Wu, Guoqing Pei, Dongya Chu, Yuting Wu, Han Gu, Siyu Wu, Chenzhuo Wang, Wanlai Zhu, Hai Zhou, Dongxia Hu

**Affiliations:** 1 Laser Fusion Research Center, China Academy of Engineer Physics, Mianyang 621900, China; wuhonghong21@gscaep.ac.cn (H.W.); ytwu_livie@163.com (Y.W.); guhan@njust.edu.cn (H.G.); siyuwuedu@163.com (S.W.); wcz2121523028@163.com (C.W.); wanlai@mails.swust.edu.cn (W.Z.); a687097@163.com (H.Z.); dongxia.hu@163.com (D.H.); 2 School of Mechanical Engineer, Tsinghua University, Beijing 100084, China; chudy15@163.com

**Keywords:** divergent light, energy distribution, detuning angle, error analysis

## Abstract

In this paper, we investigate the theory of energy distribution when divergent light undergoes harmonic conversion in KDP crystals, and based on this theory, we design and construct a precision measuring instrument for the detuning angle of (KDP) Crystals (MIDC). The device can obtain the detuning angle of the crystal by a single measurement with an average measurement error of 72.78 urad. At the same time, it also has the function of scanning the full aperture of the crystals. Using the MIDC, it is possible to quickly measure the KDP crystal at a single point and quickly scan the crystal detuning angle at full aperture. In addition, we conduct a theoretical study on the variation of detuning angle caused by gravity-influencing factors under online conditions, propose an optimization formula for the offline measurement results of detuning angle, and calculate the optimized values of detuning angle for two kinds of crystals under 45° online conditions. We finally study the error source of the MIDC device, analyze the trend of the influence of positioning errors of the crystal and optical elements on the detuning angle measurement results, and provide theoretical support for the error monitoring and correction of MIDC.

## 1. Introduction

Inertial Confinement Fusion (ICF), as an essential research direction for future energy applications, is the focus of nuclear fusion research in countries worldwide. Facilities such as the National Ignition Facility (NIF) in the U.S. [1,2,3,4,5], the Laser Megajoule (IMJ) in France [6], the 100 kilo-Joule laser facility in China [7], and the Gekko-XII in Japan [8] are all dedicated to the use of high-powered laser devices to drive fusion ignition; the NIF device has already realized a net energy gain [9].

To increase the absorption of laser energy by the target pellet it is necessary to convert the frequency of the laser by employing a frequency converter consisting of a Second Harmonic Generator (SHG) and a Third Harmonic Generator (THG), which converts the wavelength of the laser light from 1053 nm to 351 nm [10]. The large aperture KDP crystals (molecular formula: KH_2_PO_4_) are critical in the harmonic conversion process. Due to the unique properties of KDP crystals, the optimal harmonic conversion efficiency of KDP crystals can be achieved only when the incident direction of the laser is at a particular angle to the optical axis of the crystal. At this time, the angle between the crystal’s optical axis and the beam’s wave vector is called the optimal phase matching angle. In order to ensure that the optimal harmonic conversion efficiency can be achieved when the laser is incident on the crystal perpendicularly, the crystal was grown and processed in the direction that the angle between the optical axis and the surface normal is the optimal phase matching angle. However, due to the large aperture requirement of the crystal, as well as the growth and processing errors, the crystal does not reach the optimal phase matching angle. The error of the optical axis deviation from the optimal phase matching angle is called the detuning angle [11,12,13]. The detuning angle will cause the crystal to fail to obtain a better harmonic conversion efficiency. C.E. Barker et al. showed that when the SHG crystal deviates from the optimal phase-matching angle for 40 urad, or the THG crystal deviates from 120 urad, it will lead to a 5% decrease in the conversion efficiency of the harmonically-converted 351 nm beam. The decreasing trend is more and more evident with the increase of the error [14].

In order to achieve a high harmonic conversion efficiency when the crystal works online, the crystal needs to undergo a substantial amount of online adjustment after installation to keep the crystal in the best phase matching attitude. This process wastes lots of time and resources. Therefore, currently, all countries pre-measure the detuning angle of the crystal when it is offline and adjust the online attitude of the crystal with the measurement results to obtain a better harmonic conversion efficiency. Early in 1998, the Lawrence Livermore National Laboratory (LLNL) in the United States completed the development of the Crystal Alignment Checking Equipment (CAVE), which scans the crystals with a small-diameter probe laser to measure the distribution of the phase-matching angle at each point of the crystals, to monitor the inhomogeneity of the amount of phase mismatch in the large-diameter crystals [15,16]. In 2010, Kong et al. from the Shanghai Institute of Laser and Plasma Research, Chinese Academy of Engineering Physics (CAEP) used a nanosecond pulsed laser with a beam aperture of about 5 mm and a power density of 1-2 GW/cm^2^ to scan the crystal. This measures the optimal phase-matching angle at each point of the crystal and takes the average as a way of reducing the effects of the crystal’s refractive index non-uniformity and the inconsistency of processed shapes [17]. The Crystal Phase-matching angle Metrology Instrument (CPMI), designed by Pei et al. from the Laser Fusion Research Center of the Chinese Academy of Engineering Physics (CAEP) in 2018, used an Nd: YLF laser as the light source and reflected the first-instrumented laser light into the crystal for phase-matching angle measurements through a mirror fixed to a combined *X*-*Z* displacement stage, and controlled the stage to change the scanning position as a means of measuring the phase-matching distributions of the crystal at various points [18,19].

Each of these devices uses energy meters to monitor laser energy and calculate the harmonic conversion efficiency of the KDP crystal. This method requires fitting the harmonic conversion efficiency curve by changing the attitude of the crystal to find the position of maximum efficiency. Although the device uses a probe laser as the energy source, multiple laser shots are required to fit the energy efficiency curve, which is time-consuming and has high measurement errors.

Meanwhile, each crystal is placed at a particular angle due to the different transmission ways of the online optical path. In contrast, the crystal is vertical during the offline measurement. Moreover, due to the large aperture, thin thickness, and small elastic modulus of KDP crystals, the deformation of the crystals at different angles under the effect of gravity leads to inconsistency between the offline measurement results and the detuning angle of the online installation [20]. All countries have optimized crystal clamping solutions, such as using four-corner support, four sides fixed, side multi-point clamping combined with the front three-point low-stress clamping, gluing, and so on. However, the crystals still have small deformations which have some influence on their detuning angle [21].

In this paper, we propose a fast measurement method of the detuning angle of KDP crystals using divergent light as the energy source, under which the crystals can realize single-point fast measurement and multi-point fast scanning. At the same time, we study the relationship between the detuning angle changes under the online condition of the crystal to measure and optimize the detuning angle of the crystal in the online state, and to reduce the error and the number of times of the online adjustment of the crystal. Finally, we study the error sources of the MIDC device and analyze the influence of several error sources on the device’s measurement results so as to monitor the error of the MIDC.

## 2. Theory

### 2.1. Measurement Theory

The physical essence of phase matching is to make the harmonic conversion light stimulated at various points along the crystal have the same phase as the fundamental frequency light when they propagate to the exit surface. In this way, they can interfere and enhance each other, thus achieving harmonic conversion. To realize the effective conversion of nonlinear wavelengths, the conservation of photon energy and the conservation of photon momentum need to be observed simultaneously in the nonlinear optical process, and the equation for the conservation of photon momentum is known as the constraints of “phase matching”, which is:

(1) $$\Delta k=k_{3}-k_{1}-k_{2}=\frac{\omega_{3}}{c}n_{3}i_{3}-\frac{\omega_{1}}{c}n_{1}i_{1}-\frac{\omega_{2}}{c}n_{2}i_{2}=0.$$

where *k*_j_, *ω*_j_, *n*_j_, and *i*_j_ are the beam wavevector, beam frequency, refractive index of the beam in the crystal, and wavevector direction, respectively (j = 1, 2, 3).

According to the definition of harmonic conversion efficiency:

(2) $$\eta =\frac{P^{2\omega }}{p^{\omega }}$$

where *P^2^^ω^* is the second harmonic beam energy and *P^ω^* is the fundamental frequency beam energy.

Under the small signal approximation, we can theoretically derive:

(3) $$\eta \propto \frac{\sin^{2}(L\cdot \Delta k/2)}{{\left(L\cdot \Delta k/2\right)}^{2}}$$

where *L* is the through-light length of the harmonic converter crystal.

**Figure 1:**
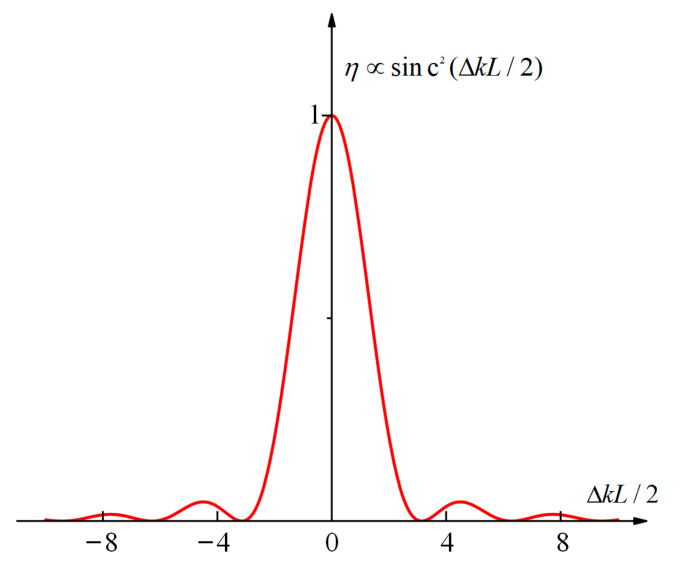
Harmonic conversion efficiency for different Δ*k*.

Hence, the harmonic conversion efficiency curve of the crystal for laser light can be obtained, as shown in Figure 1.

**Figure 2:**
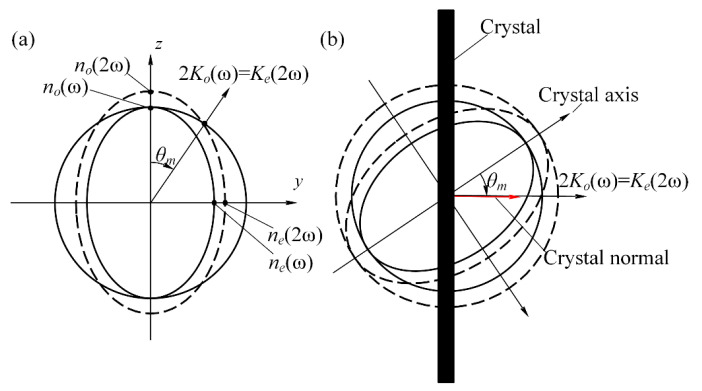
KDP crystal phase-matching schematic (**a**) and crystal principal optical axis (**b**).

During the harmonic conversion, a 1053 nm linearly polarized Gaussian beam (*o*-light) is used as the fundamental frequency beam. The second harmonic (527 nm) and the third harmonic (351 nm) are generated in the crystal by way of type I matching (*o + o = e*) or type II matching (*e + o = e*). Due to the nonlinear effect of KDP crystals, the crystals have different refractive indices for ordinary light (*n_o_*) and extraordinary light (*n_e_*), and the refractive index change can be visualized by the refractive index ellipsoid (e.g., Figure 2a). The difference in the refractive indices of the beams leads to a change in the phase difference of the beams of each wavelength, which in turn leads to a change in the harmonic conversion efficiency. As shown in Figure 2b, the crystal is generally processed to make the angle *θ* between the surface normal of the crystal and the optical axis the optimal phase matching angle *θ_m_*. At this time, the beam incident perpendicular to the surface of the crystal can achieve the optimal harmonic conversion efficiency. However, due to the processing error of the crystal and the requirement of a large aperture, *θ* and *θ_m_* are usually unequal, which leads to the detuned attitude of the crystal, and better harmonic conversion efficiency cannot be obtained. The difference between *θ* and *θ_m_* is the detuning angle Δ*θ_m_*. Therefore, measuring the detuning angle of the crystal with high precision is necessary to ensure that it can be tuned to the optimal phase-matching state directly when it is working online.

**Figure 3:**
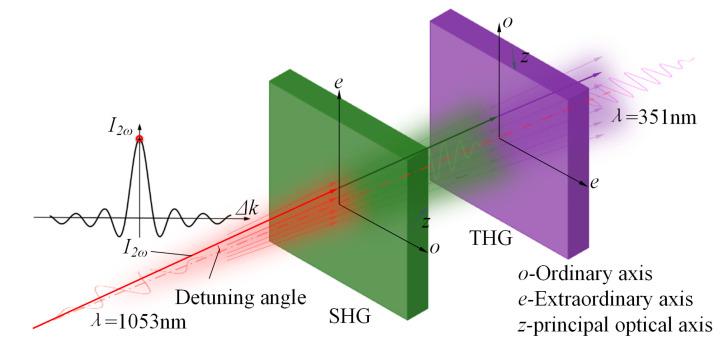
Harmonic conversion process of divergent light.

**Figure 4:**
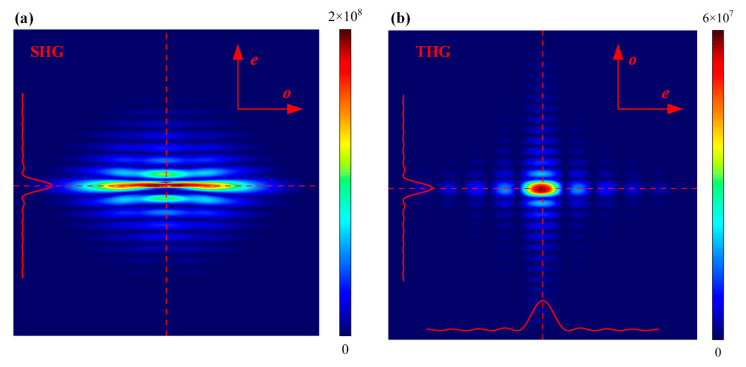
(**a**) is the energy distribution after second harmonic conversion, (**b**) is the energy distribution after third harmonic conversion.

During the offline measurement of crystals, if the harmonic conversion is performed with a collimated beam, the energy of the beam after harmonic conversion is uniformly distributed due to all points in the beam having the same value, so the energy can only be received by energy meters, and the harmonic conversion efficiency curve of the crystal is fitted by changing the crystal attitude, so as to obtain the crystal attitude at the highest efficiency of the harmonic conversion and to calculate the detuning angle. This method takes a long time to measure and has a significant error. As shown in Figure 3, we placed a divergent lens in front of the crystal to increase the beam’s divergence angle. Each component of the divergent beam can separately perform the harmonic conversion in the crystal, and the harmonic conversion efficiency of each component is varied, so that the energy distribution after the crystal in the direction of the e-axis will also have the same change as that of the energy conversion efficiency curve in general. Since the e axis of the second harmonic generation (SHG) crystals and the third harmonic generation (THG) crystals are perpendicular to each other, the direction of change of their energies is spaced 90° apart. We obtained the energy distribution after the crystal by simulation, as shown in Figure 4.

When the crystal is rotated around the *o*-axis at a little angle, the variation of the angle between the optical axis of the crystal and the beam will cause the component with the highest harmonic conversion efficiency in the divergent light to change, resulting in the energy distribution after the crystal shifting. The crystal’s rotation angle and the shift’s distance are linearly related. Hence, we can rotate a standard crystal, in which the detuning angle is known, and record the position of the energy peak after the crystal at different angles to obtain a linear relationship between the rotation angle of the standard crystal and the energy distribution. We can use this linear relationship and the energy distribution of the unknown crystal, which is at a specific angle to the beam (generally, the beam is perpendicular to the crystal surface), to calculate the detuning angle of the unknown crystal.

### 2.2. Optimization Theory for Detuning Angle Measurement Result

Since the KDP crystal is in a vertical position during offline measurements, the effect of gravitational deformation on the detuning angle of the crystal is minimal. When the crystal is installed in the final optical assembly, the crystal needs to be placed in different attitudes due to the various transmission directions of the beam. However, the crystal is characterized by a large aperture, thin thickness, and small modulus of elasticity, and the crystal will have a surface change when subject to gravity, which causes the detuning angle of the crystal at each point in the online condition to differ from that of the offline measurement value.

Since the width of a large aperture crystal is much larger than its thickness, the crystal can be regarded as an elastic thin plate. When analyzing the change in the surface shape of the crystal, the small deflection equation for thin plate bending in elastic mechanics can be used to solve the problem:

(4) $$q=D_{1}\frac{{𝜕}^{4}\omega }{𝜕x^{4}}+2D_{3}\frac{{𝜕}^{4}\omega }{𝜕x^{2}y^{2}}+D_{2}\frac{{𝜕}^{4}\omega }{𝜕y^{4}}$$

where *ω* is the deflection, *q* is the gravitational component, and *D*_1_ *= D*_11_, *D*_3_ *= D*_12_ *+* 2*D*_66_, and *D*_2_ *= D*_66_, all are stiffness matrix elements for each anisotropic crystal:

(5) $$\left[D\right]=\left[\begin{array}{cccccc} D_{11} & D_{12} & D_{13} & 0 & 0 & 0\\ D_{12} & D_{11} & D_{13} & 0 & 0 & 0\\ D_{13} & D_{13} & D_{33} & 0 & 0 & 0\\ 0 & 0 & 0 & D_{44} & 0 & 0\\ 0 & 0 & 0 & 0 & D_{44} & 0\\ 0 & 0 & 0 & 0 & 0 & D_{66} \end{array}\right]$$

where *D*_11_ = 71.2 GPa, *D*_12_ = −6.27 GPa, *D*_13_ = 14.94 GPa, *D*_33_ = 56.4GPa, *D*_44_ = 12.48 GPa, and *D*_66_ = 6.21 GPa.

By solving Equation (4), we can obtain the deformation equation of the crystal under different gravitational conditions, and the directional derivative of this equation in the direction of crystal rotation is the detuning angle error caused by gravity. Here, it is assumed that the SHG crystal rotates around the *x*-axis and the THG crystal rotates around the *y*-axis.

(6) $$\left\{\begin{array}{c} \Delta \theta_{m(SHG)}^{G}=grad_{x}(x,y)=\frac{𝜕\omega }{𝜕x}\\ \Delta \theta_{m(THG)}^{G}=grad_{y}(x,y)=\frac{𝜕\omega }{𝜕y} \end{array}\right.$$

Meanwhile, the change of relative harmonic conversion efficiency caused by the detuning angle error at each node can be calculated by Equation (3). We take the relative harmonic conversion efficiency at each point as the weight, and make a weighted average calculation of the detuning angle error to obtain the detuning angle error of the crystal under the influence of gravity:

(7) $$\left\{\begin{array}{c} \Delta {\bar{\theta }}_{m(SHG)}^{G}=\frac{1}{X_{\max}\cdot Y_{\max}}\int_{0}^{X_{\max}}\int_{0}^{Y_{\max}}\eta (x,y)\cdot grad_{x}(x,y)dxdy=\frac{1}{X_{\max}\cdot Y_{\max}}\int_{0}^{X_{\max}}\int_{0}^{Y_{\max}}\eta (x,y)\frac{𝜕\omega }{𝜕x}dxdy\\ \Delta {\bar{\theta }}_{m(THG)}^{G}=\frac{1}{X_{\max}\cdot Y_{\max}}\int_{0}^{X_{\max}}\int_{0}^{Y_{\max}}\eta (x,y)\cdot grad_{y}(x,y)dxdy=\frac{1}{X_{\max}\cdot Y_{\max}}\int_{0}^{X_{\max}}\int_{0}^{Y_{\max}}\eta (x,y)\frac{𝜕\omega }{𝜕y}dxdy \end{array}\right.$$

This error optimizes the detuning angle measurements of the offline measurement system so that the offline measurements tend to match the online angle of the crystal.

## 3. Design of MIDC

**Figure 5:**
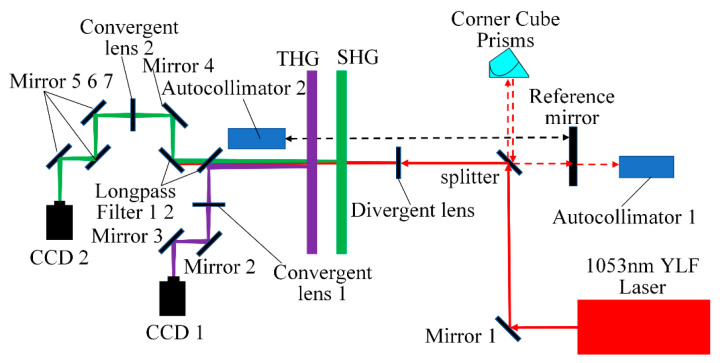
The optical path of MIDC.

The MIDC is designed to accurately measure the detuning angle of large aperture KDP crystals. The device employs CCD cameras to monitor the energy distribution after harmonic conversion, and the energy intensity is displayed in the imaging as the size of the pixel point gray value. Its optical path diagram is shown in Figure 5.

An Nd: YLF probe laser is used as the laser source, and the beam is reflected through reflector 1 and then divided into two parts by the splitter. One part of the beam is used for optical path collimation, and the other part passes through a divergent lens to become diverging light. The divergent beam is transmitted to the SHG and THG crystals for harmonic conversion. The harmonic conversion beam contains a fundamental frequency beam (1053 nm), a second harmonic beam (527 nm), and a third harmonic beam (351 nm). The third harmonic beam will be reflected by long pass filter 1 and converged through convergent lens 1 for beam contraction to ensure that the CCD1 can sufficiently receive the energy distribution of the third harmonic beam. The remaining beam is separated by long pass filter 2, the 1053 nm fundamental frequency beam passes directly through the long wavelength pass filter, the second harmonic beam is reflected by long wavelength pass filter 2 and mirror 4, and through convergent lens 2 for convergence, and then the second harmonic beam is finally transmitted through mirrors 5, 6, and 7 to the CCD2 for imaging. The detuning angle of crystals is obtained by calculating the position of the pixel point with the most significant gray value in the CCD imaging. The main parameters of the device and the distances between the components are shown in Table 1 and Table 2.

**Figure 6:**
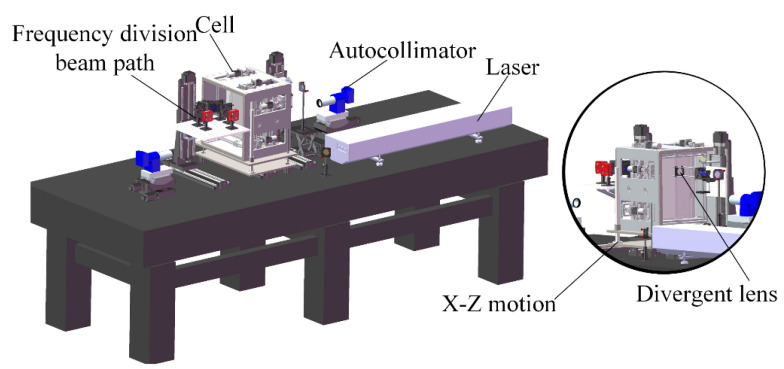
The structure of MIDC.

The structure of the MIDC is shown in Figure 6. The device mainly consists of a laser, a harmonic conversion cell, an *X*-*Z* motion mechanism, a split-frequency optical path, and a collimation module. The harmonic conversion cell is reserved with loading space for KDP crystals, which facilitates the loading and replacement of KDP crystals. It is also equipped with two sets of pitch-deflection motors to drive the KDP crystal angle for adjustment, and the rotational accuracy of the motors is 1.8urad. A high-precision motorized displacement stage provides the *X*-*Z* motion mechanism for *X*-direction operation, and two guide columns and linear stepping motors provide *Z*-direction movement. The split-frequency optical path module is an integrated module that receives and separates the mixed-frequency beam emitted from the THG. Optical components, such as long-wavelength pass filters, convergent lenses, and mirrors, are integrated into a cage system to split the mixed-frequency beam and monitor the energy.

**Figure 7:**
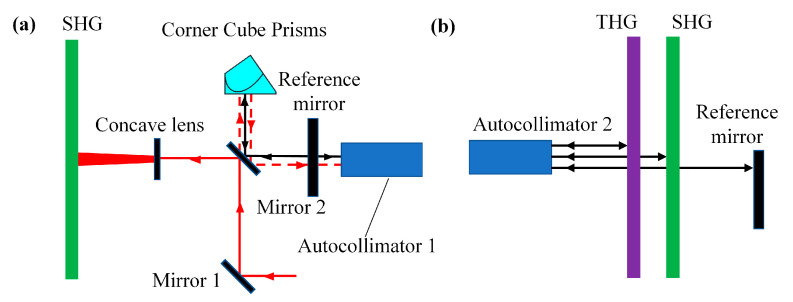
(**a**) shows the optical path collimation process of MIDC and (**b**) shows the crystal collimation process of MIDC.

The function of the collimation module is to make the laser’s incidence perpendicularly in the KDP crystal. The collimation process includes the optical path collimation and the crystal collimation. As shown in Figure 7a, one part of the beam is transmitted to the corner cone prism from the splitter. The corner cone prism can make any direction of the incident beam to the opposite direction of propagation to ensure the beam’s parallelism. The beam propagates in the opposite direction and is reflected by the beam splitter to the autocollimator 1; simultaneously, the autocollimator 1 launches a crosshair and is reflected to the autocollimator 1 by the reference mirror. By adjusting the angle between the reference mirror and the autocollimator 1, the crosshair launched by the autocollimator and returned to itself will coincide with the laser beam, i.e., the collimation of the optical path is completed. At this time, the laser is perpendicular to the reference mirror.

The schematic diagram of the collimation of the crystal is shown in Figure 7b, adjusting the angle of the autocollimator 2 so that the crosshair launched by the autocollimator is perpendicular to the reference mirror, and then adjusting the SHG and THG crystal through the motor so that the crosshairs launched by the autocollimator is reflected back to the autocollimator 2 from the crystals and ensuring that the launched and returned crosshairs are overlapped, which means that the collimation process of the crystal is completed. At this time, the reference mirror and the two crystals are perpendicular to the beam launched by the autocollimator 2, so that it can be known that the crystals are perpendicular to the beam launched by the laser.

When we fit the linear relationship between the detuning angle and the energy distribution, the sample crystal is clamped in the harmonic conversion cell, the crystal is rotated around the o-axis by the motor-driven crystal frame, and the rotation angle of the crystal is monitored by autocollimator 2. At the same time, the energy distribution in the CCD is recorded to complete the fitting of the linear relationship. When measuring the crystal with an unknown detuning angle, the crystal is adjusted to the perpendicular of the crosshair emitted by the autocollimator 2, and the energy distribution in the CCD is recorded, so that the detuning angle of the unknown crystal can be obtained through the position of this energy distribution in the fitted linear relationship.

## 4. Experiments

### 4.1. Measurement Method

**Figure 8:**
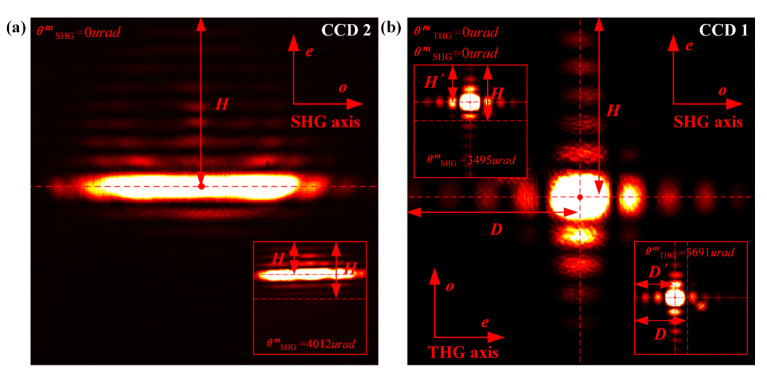
Second harmonic energy distribution (**a**) and third harmonic energy distribution (**b**) in CCD camera.

When the MIDC is loaded with only SHG crystal, the harmonic conversion cell can only convert the fundamental frequency beam into the second harmonic beam. Meanwhile, the energy distribution only exists in the CCD2 camera that monitors the second harmonic, as shown in Figure 8a. When two kinds of crystals are loaded in the harmonic conversion cell, the second and third harmonics exist simultaneously after the crystals, and CCD1 and CCD2 can observe the energy distributions of 351 nm and 527 nm beams, respectively. However, due to part of the second-harmonic energy absorbed during the third harmonic conversion of the THG crystal, an amount of the second-harmonic energy is missing in the imaging, and the missing energy corresponds to the higher position of the third harmonic energy distribution. At this time, the imaging in CCD2 is not suitable for the measurement of the detuning angle of the SHG crystal. However, the energy distribution of the third harmonic in the *e*-axis direction of the SHG crystal in CCD1 is distinct and corresponds to the missing energy of the second harmonic. Therefore, this energy distribution can be used to measure the detuning angle of SHG and THG crystals (Figure 8b).

#### 4.1.1. Linear Relationship Fit

When using divergent light to measure the detuning angle of a KDP crystal offline, it is vital to fit a linear relationship between the detuning angle of the standard crystal and the energy peak. In the detuning angle measurement of an unknown crystal, we will record the position of its energy peak and find the corresponding detuning angle in this fitting relationship. Therefore, the quality of the fitting relationship will significantly affect the accuracy of detuning angle measurements for unknown crystals. We analyze the quality of the fitting relationship by the coefficient of determination (R-square) of the fitting relationship, and the errors between each fitting point and the fitting relationship.

**Figure 9:**
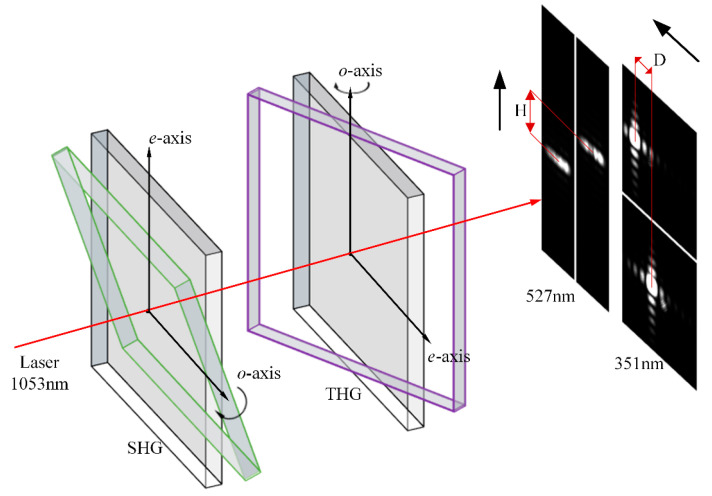
Schematic of the fitting process.

We fit the linear relationship between the position of the energy distribution and the crystal detuning angle for SHG and THG standard crystals. The MIDC drives the crystal to rotate around the o-axis at a fixed angle and compiles the algorithm to find the center of the brightest spot in the camera as the highest energy distribution point. The position of the energy peak changes with the angle of rotation (H for the SHG crystal, D for the THG crystal), and the fitting process is shown in Figure 9.

**Figure 10:**
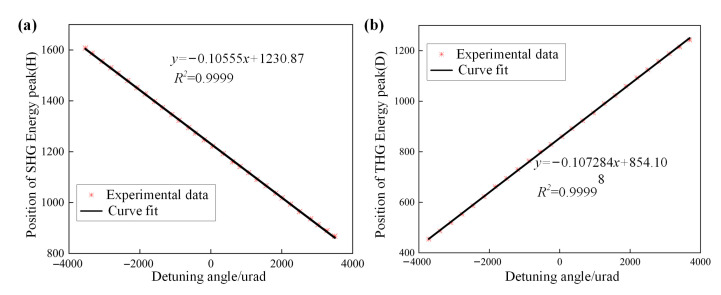
(**a**) SHG and (**b**) THG linear relationship between detuning angle and peak energy position.

**Figure 11:**
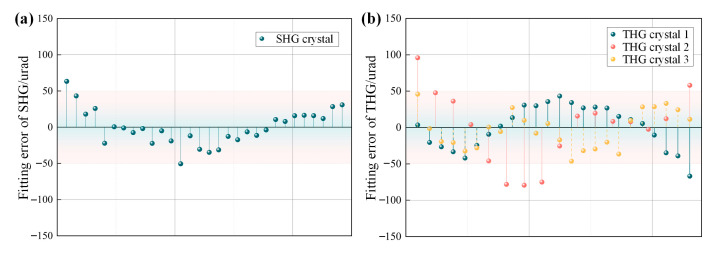
(**a**) is the errors in fitting linear relationships of SHG and (**b**) is the errors in fitting linear relationships of THG.

The linear relationship between the crystal detuning angle and the position of the energy peak is shown in Figure 10, and the determination coefficients of the linear relationship fit between the SHG standard crystal and the THG standard crystal are more than 0.9999, with an excellent linear regression. Meanwhile, the errors between each fitting point and the fitting line are shown in Figure 11; the maximum fitting error of the linear relationship for the detuning angle of SHG crystals is 63.3 urad, and the fitting maximum mistake of THG crystals is 95.9 urad. The errors between most of the measurement points at the terminals of the fitting relationship and the fitted relationship are controlled to be less than 50 urad. This means that when the linear relationship is used to measure the detuning angle of the unknown crystals, the measurement error due to the fitting method is controlled between 50 urad.

#### 4.1.2. Multi-Point Measuring Accuracy

Due to the crystal’s large aperture, it is impossible to ensure the consistency of the detuning angle at each point of the crystal during growth and processing, so it is necessary to measure the detuning angle of the crystal at the full aperture. The MIDC device scans the full aperture of the crystal by driving the crystal in the *X*-*Z* plane using motors, without changing the optical path. It averages the results of the measurements at each point as a measurement of the detuning angle of the crystal. However, during the complete aperture scanning process, it is impossible to predict whether the variation in the detuning angle measurement at each point is due to the growth process or the measurement error of the MIDC during the multi-point measurement. Therefore, we used a standard crystal with good planarity to measure the multi-point accuracy of the MIDC. We randomly moved the standard crystal in the *X*-*Z* plane and measured the detuning angles at several points.

**Figure 12:**
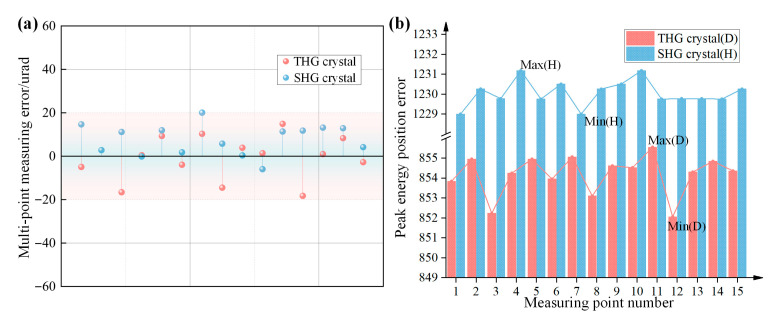
(**a**) shows the multi-point measurement accuracy of MIDC and (**b**) shows the energy peak position hopping errors in multi-point measurement.

By comparing the detuning angle measurements at each point of the standard crystal with the standard value of this crystal, we obtained the imaging and measurement error interval of MIDC for multi-point measurements of crystal detuning angle, as shown in Figure 12. The detuning angle measurement errors of MIDC are stabilized to be within 20 urad at the full aperture of the same crystal, and the energy peak position hopping errors are within 3.5-pixel points. Therefore, when scanning an unknown crystal at full aperture, if the variation range of the detuning angle of all crystal points is smaller than this error range, we can be sure that MIDC causes the variation.

#### 4.1.3. Repeatable Measurement Accuracy

To ensure the stability and repeatability of the crystal detuning angle measurement by the MIDC over a long period, the repeatability of the MIDC needs to be measured. We define the repeatability of the MIDC to include both temporal and spatial stability. Temporal stability refers to whether the measurement error of the device is too large for the same crystal after an extended period, and spatial stability refers to whether the measurement error is too large when the crystal undergoes attitude changes and then returns to the original attitude for measurement.

**Figure 13:**
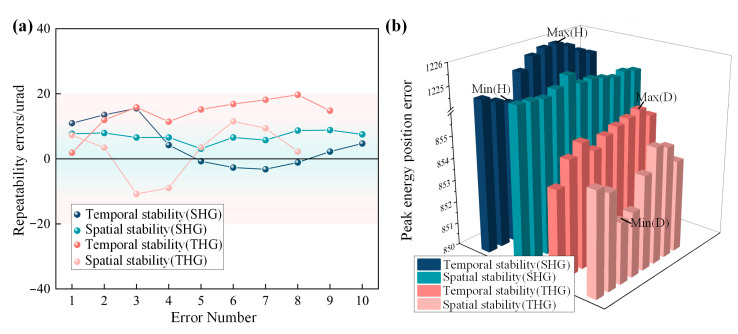
(**a**) shows the repeatability of MIDC and (**b**) shows the energy peak position hopping.

We verified the temporal stability of the standard crystal by taking measurements every five minutes with a consistent crystal attitude, and we also verified its spatial stability by motor-driving the crystal to change its attitude randomly and then adjusting it to the original attitude and taking a measurement. Figure 13 shows the repeated accuracy measurement results of the MIDC. The repeated measurement accuracy of the MIDC is stabilized within 20 urad, with a maximum energy peak position hopping error of 2.2 pixels. It can be seen that the MIDC has good measurement stability for crystals in a long period range.

#### 4.1.4. Unknown Crystal Detuning Angle Measurements

We measured KDP crystals with unknown detuning angles using the MIDC under the same ambient temperature conditions as online. We also measured the detuning angle of these unknown crystals with a previous-generation offline measurement system (CPMI). The CPMI device uses a collimated beam to measure the crystal detuning angle. A beam-splitting prism is placed at the back of the crystal to separate the beams of different wavelengths, and an energy meter is used to monitor the energy and calculate the harmonic conversion efficiency. It searches for the detuning angle by changing the crystal attitude.

**Figure 14:**
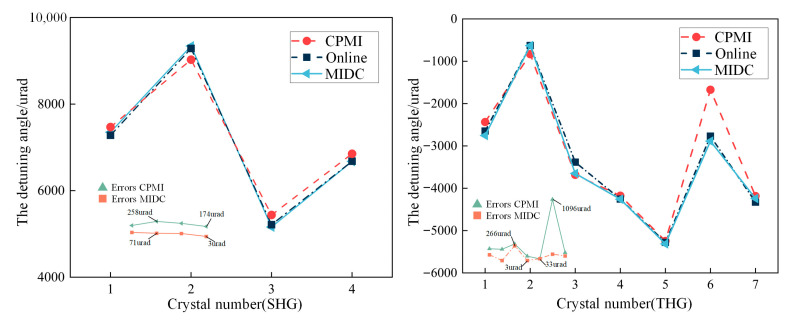
Measurements of the detuning angle of unknown crystals.

As shown in Figure 14, we took the angle at which the crystals reached the optimal harmonic conversion efficiency in the terminal optics assembly during online commissioning as the reference, and compared the measurement results of the MIDC device with those of the CPMI device. The measurement results show that the maximum measurement errors of MIDC for SHG and THG crystals are 71 urad and 266 urad, respectively. The mean and standard deviation of the measurement errors are 72.78 urad and 75.78 urad, respectively, which is 73% higher than that of the offline measurement system’s previous generation in terms of accuracy and stability. Therefore, after the offline measurement of the crystal, we only need to tune the crystal online 1–2 times to obtain a high harmonic conversion efficiency.

### 4.2. Optimization of Measurement Results

Due to the difficulty of solving the analytical solution for the crystal deflection, we use the finite element method for the problem. At this time, the information about the deformation of KDP crystals can be expressed in the form of a matrix:

(8) $$\omega (x,y)=\left[\begin{array}{cccc} \omega_{11} & \omega_{12} & \ldots & \omega_{1j}\\ \omega_{21} & \omega_{22} & & \\ \ldots & & \ldots & \\ \omega_{i1} & \omega_{i2} & & \omega_{ij} \end{array}\right],i=1,2,\ldots ,N,j=1,2,\ldots ,M$$

where *N* and *M* are the grid sampling densities. At this time, the deformation gradient matrix of the crystal is:

(9) $$grad_{x}(x,y)=\left[\begin{array}{cccc} g_{11}^{x} & g_{12}^{x} & \ldots & g_{1j}^{x}\\ g_{21}^{x} & g_{22}^{x} & & \\ \ldots & & \ldots & \\ g_{i1}^{x} & g_{i2}^{x} & & g_{ij}^{x} \end{array}\right],i=1,2,\ldots ,N-1,j=1,2,\ldots ,M-1$$

(10) $$grad_{y}(x,y)=\left[\begin{array}{cccc} g_{11}^{y} & g_{12}^{y} & \ldots & g_{1j}^{y}\\ g_{21}^{y} & g_{22}^{y} & & \\ \ldots & & \ldots & \\ g_{i1}^{y} & g_{i2}^{y} & & g_{ij}^{y} \end{array}\right],i=1,2,\ldots ,N-1,j=1,2,\ldots ,M-1$$

The following equation can find the elements in its deformation gradient matrix:

(11) $$\left\{\begin{array}{c} g^{x}{\;}_{i,j}=\frac{\omega (i+1,j)-\omega (i-1,j)}{2\delta_{x}}\\ g^{y}{\;}_{i,j}=\frac{\omega (i,j+1)-\omega (i,j-1)}{2_{\delta y}} \end{array}\right.$$

Thus, the detuning angle error of the crystal under the influence of gravity is:

(12) $$\left\{\begin{array}{c} \Delta {\bar{\theta }}_{m(SHG)}^{G}=\frac{1}{\left(N-2)(M-2\right)}\sum_{i=1}^{N-2}\sum_{j=1}^{M-2}\eta (i,j)\cdot \frac{\omega (i+1,j)-\omega (i-1,j)}{2\delta_{x}}\\ \Delta {\bar{\theta }}_{m(THG)}^{G}=\frac{1}{\left(N-2)(M-2\right)}\sum_{i=1}^{N-2}\sum_{j=1}^{M-2}\eta (i,j)\cdot \frac{\omega (i,j+1)-\omega (i,j-1)}{2\delta_{y}} \end{array}\right.$$

**Figure 15:**
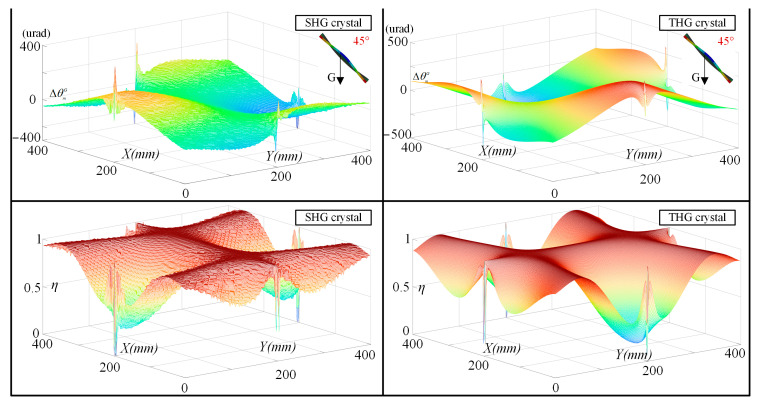
The simulation analysis on the variation of detuning angle and harmonic conversion efficiency under the influence of gravity at each point of the crystal.

Under the conditions affected by gravity, we simulated and analyzed the deformation of the crystals using a four-point clamping scheme. SHG crystals (410 × 410 × 12 mm) and THG crystals (410 × 410 × 9 mm) were modeled separately under the condition that the surface normals differed from the direction of gravity by 45°, and fixed constraints were applied in the middle of the four sides of the upper and lower surfaces of the crystals. After obtaining the face shape variations matrix of the crystals, we calculated the variation values of the detuning angle and the relative conversion efficiency of the crystals by Equations (3) and (8)–(11), respectively. As shown in Figure 15, under the condition of 45° tilt, the SHG crystal varies within ±400 urad of its detuning angle due to the effect of gravitational deformation, and the THG crystal goes within ±380 urad. We bring the relative harmonic conversion efficiency and detuning angle error matrix into Equation (12) to obtain the detuning angle measurements that need to be optimized for the crystal under the influence of gravity. The theoretically optimized value for the SHG crystal tilted at 45° is −0.2868 urad, and 0.031 urad for the THG crystal.

**Figure 16:**
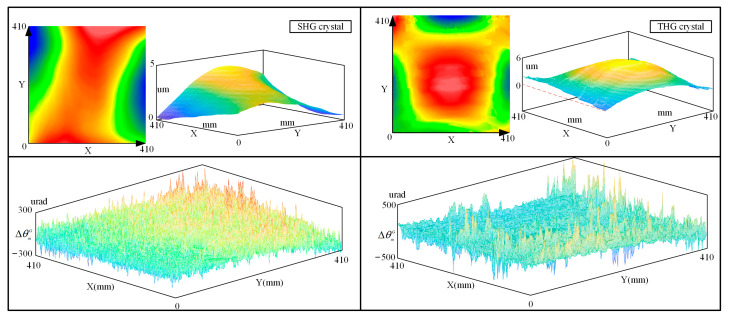
Real face shape and detuning angle variation of crystals in the online attitude.

The actual crystals also have deformation from other factors, such as clamping force in the process of gravity. We use the interferometer to scan the face shape of several unknown crystals under gravity conditions, and the value of the change of their detuning angle is calculated. As shown in Figure 16, we scan the face shape of an unknown SHG crystal and a THG crystal, respectively, and calculate the change of detuning angle at each point. After calculating Equation (12), we determined the optimized detuning angle error values under gravity for these two crystals as 11.59 urad and −3.5077 urad, respectively.

## 5. Error Source Analysis of MIDC

When measuring the detuning angle of a crystal with the MIDC, the primary error sources are the positioning error of the crystal and the error generated by the relative position of the optical elements. When the crystal is rotating around the *o*-axis, there is also a slight movement around the *e*-axis for detuning angle measurement. At the same time, the relative positions of the optical elements in the MIDC can be shifted due to vibration, resulting in changes in the image in the CCD. In the following, we will analyze the trend of these factors on the error of energy distribution in the CCD.

### 5.1. Effect of Crystal Positioning Error on Detuning Angle

By rotating the crystals, we found that the energy distribution changes significantly when the SHG crystals are rotated around the e-axis. At the same time, the THG crystals revolve around the e-axis with a tiny effect on the energy distribution. Therefore, we investigate the impact of crystal localization on the detuning angle error based on SHG crystals.

**Figure 17:**
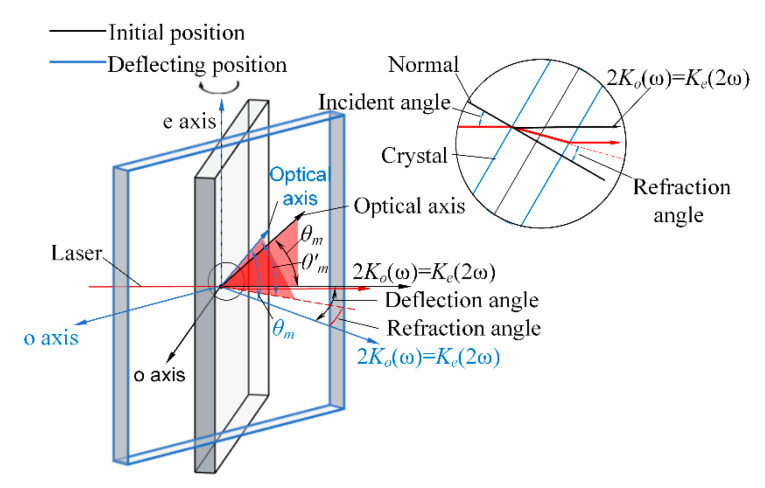
Crystal axis direction and beam propagation direction during crystal deflecting.

**Figure 18:**
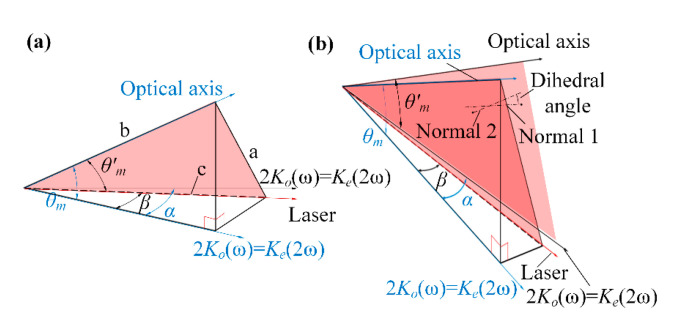
(**a**) shows the plane *S* and (**b**) shows the transformation of plane *S* and each parameter.

When the crystal is rotated around the e-axis, the variation of *θ* is shown in Figure 17. Since the SHG crystal adopts type I harmonic conversion (*o + o = e*), the refractive index of the beam incident into the crystal is consistent in all directions. As shown in Figure 18a, when the deflection angle of the SHG crystal around the e-axis is *α*, and the refractive angle of the beam is *β*, the relationship between *θ* and *α*, *β*, and *θ_m_* at this time can be found by Equation (13):

(13) $$\left\{\begin{array}{l} n\cdot \sin\alpha =n_{o}(\omega )\cdot \sin\beta \\ a=\sqrt{\tan^{2}\theta_{m}+\tan^{2}\beta }\\ b=\frac{1}{\cos\theta_{m}}\\ c=\frac{1}{\cos\beta }\\ \theta =\frac{\arccos(b^{2}+c^{2}-a^{2})}{2bc} \end{array}\right.$$

In addition, there can be constructed a plane *S* between the crystal optical axis vector and the wave vector of the beam (Figure 18b), and when the crystal is deflected, the optical axis of the crystal is no longer located in the plane *S*, instead, a new plane *S’* is constructed with the wave vector. At this time, the angle between the two planes is *φ*, which rotates the energy distribution in the CCD. *φ* can be calculated by the dihedral angle formula:

(14) $$\left\{\begin{array}{l} \vec{n_{1}}=\left|\begin{array}{ccc} \vec{i} & \vec{j} & \vec{k}\\ \cos(\alpha -\beta ) & \sin(\alpha -\beta ) & 0\\ \cos\theta_{m}\cos\alpha & \cos\theta_{m}\sin\alpha & \sin\theta_{m} \end{array}\right|,\vec{n_{2}}=\left|\begin{array}{ccc} \vec{i} & \vec{j} & \vec{k}\\ 1 & 0 & 0\\ \cos\theta_{m} & 0 & \sin\theta_{m} \end{array}\right|\\ \varphi =\arccos\frac{\left|\vec{n_{1}}\cdot \vec{n_{2}}\right|}{\left|\vec{n_{1}}\right|\cdot \left|\vec{n_{2}}\right|} \end{array}\right.$$

When the crystal rotates, i.e., the crystal rotates around the laser wave vector, the detuning angle Δ*θ* remains unchanged, but φ changes, at which time the energy distribution behind the crystal rotates, and the angle of rotation is equal to the angle of its roll.

**Figure 19:**
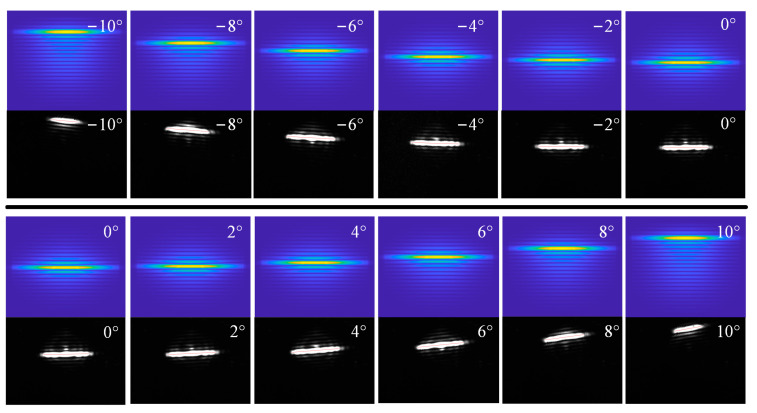
Theoretical versus actual displacement of stripes during crystal deflection.

**Figure 20:**
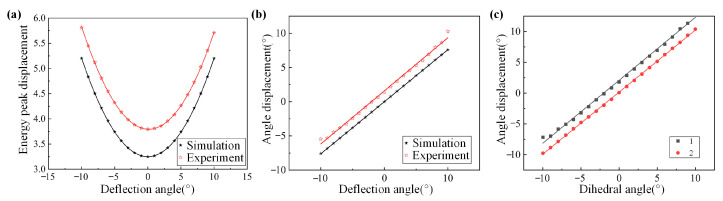
(**a**) shows the theoretical and actual displacement curves of the peak energy of a crystal in a deflected attitude, (**b**) shows the actual and theoretical angular displacements produced by the stripes when deflecting the crystal, and (**c**) where 1 is the variation of angular displacements concerning the projected angle of φ when deflecting the crystal, and 2 is the variation of angular displacements concerning the projected angle of φ when the crystal is tumbling.

We carried out theoretical simulation and experimental verification of the energy distribution of the crystal deflection angle α in the range of ±10° transformation, where α = 0° is taken when the crystal surface is found to be parallel to the laser and the clockwise direction is the positive direction. The experimental images and experimental results are shown in Figure 19 and Figure 20a, respectively. It can be seen that the energy distribution changes when α varies uniformly at ±10°. We used a quadratic function to fit the simulation and experimental results and obtained a high fitting coefficient. When the positioning error of the crystal in the direction of deflection around the *e*-axis is controlled within ±0.3423°, the measurement error of the detuning angle caused by it can be controlled within ±5 urad.

When the crystal is deflected around the *e*-axis or rolled around the wave vector, the rotation angle of the energy distribution is shown in Figure 20b,c. The relationship between the rotation angle of the energy induced by the deflection of the crystal and the deflection angle α has a high degree of linearity. In contrast, the rotation angle of the energy distribution in the case of tumbling is the angle of the tumbling. In order to stabilize the energy distribution, the positioning error of the crystal in both the deflection and tumbling directions needs to be effectively controlled.

### 5.2. Effect of Optical Element Positioning Error on Detuning Angle

When analyzing the experimental images, the size of the energy distribution is an essential factor that affects the linear relationship between the detuning angle/energy peak position. The characteristic parameters of the optics and the laser, and the positional parameters, determine the size of the energy distribution. The basic parameters of the laser and the optics have been fixed after constructing the optical path. Still, some vibrations generated during the measurement will cause the position of the optics to shift, which will lead to some errors in the measurement of the experimental results.

Since the beam launched from the laser is Gaussian, after passing through the divergent lens, the beam waist size and beam waist position are varied, thus increasing the divergence angle. Therefore, the energy distribution of the fundamental frequency light at each point in space after passing through the diverging lens can be calculated. The following is the transformation formula for a Gaussian beam passing through a diverging lens:

(15) $$\left\{\begin{array}{l} L^{\prime }=-f-\frac{(L-f)\cdot f^{2}}{{\left(L-f\right)}^{2}+Z_{R}}\\ {\omega^{\prime }}_{0}^{2}=\frac{f^{2}\omega_{0}}{{\left(L-f\right)}^{2}+Z_{R}} \end{array}\right.$$

After the harmonic conversion of the divergent light, its wavelength, amplitude, and phase will be changed, and the Gaussian beam propagation formula is no longer applicable in the spatial propagation process after the crystal, so the angular spectrum theory of diffraction is used in the spatial propagation process after the crystal to calculate the energy distribution. During the beam’s transmission after the frequency doubling of the crystal, the convergent lens imposes a phase transformation factor on the beam, so that the divergent wave becomes a convergent wave, and thus the spot is converged. The phase transformation factor of the converging lens is:

(16) $$t(x,y)=\exp\left[-j\frac{k}{2f}(x^{2}+y^{2})\right]$$

**Figure 21:**
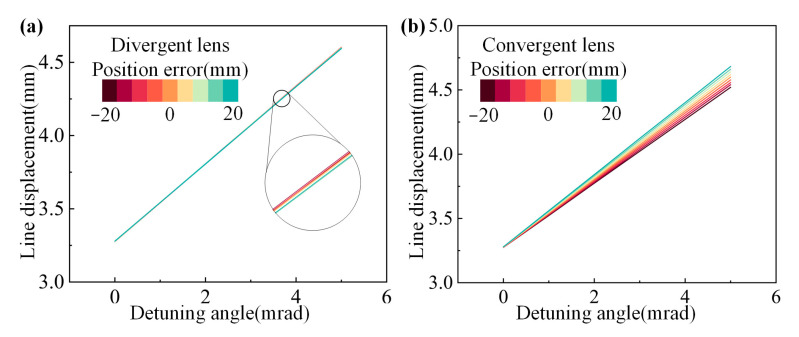
Displacement curves with different lens positions. Displacement curves with (**a**) divergent lens and (**b**) convergent lens positions.

**Figure 22:**
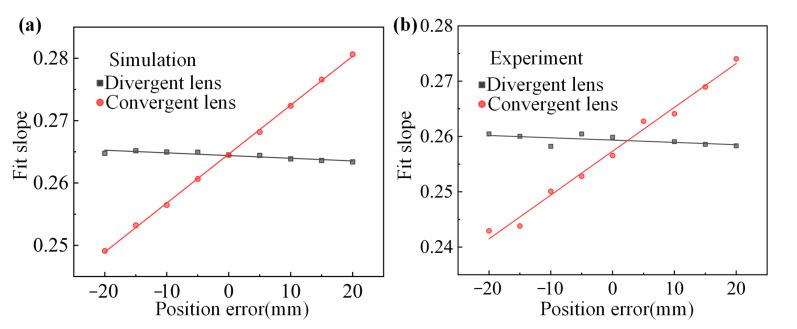
Slope change of the displacement curve. Slope change of the displacement curve ((**a**) Simulation and (**b**) Experiment).

Since only the lens changes the beam’s divergence angle during its propagation, and the positional errors of the other optical elements only change the propagation distance of the beam, the relative positional errors between each optical element can be converted to the positional error of the lens for calculation. When the lens position is varied in a certain error range, the variation of the detuning angle/peak energy displacement curve is shown in Figure 21, which shows that the position shift of the lens changes the slope of the stripe displacement curve. The trend of the stripe displacement curve with the position error of the lens is shown in Figure 22. The trend of the influence of the diverging lens on the slope of the stripe displacement curve is on the order of 10^−3^. The positional error of the diverging lens allowed by the index parameter within the measurement range of the crystal detuning angle is 18.87 mm, so the tiny displacement of the diverging lens caused by vibration and other factors is entirely within the measurement error. The influence of the converging lens on the slope of the stripe displacement curve tends to be larger, and the positional error of the converging lens allowed by the index parameter is 1.025 mm, so the positional error of the converging lens is an essential index for the error monitoring of the measurement system.

## 6. Conclusions

This paper investigates the energy distribution of divergent light undergoing harmonic conversion, and an offline measurement device for the detuning angle of KDP crystals (MIDC) is designed and constructed using divergent light as the scanning light source. The MIDC monitors the energy distribution after harmonic conversion with two CCD cameras. It solves the problem that the previous generation of measurement devices needed to measure the energy several times for each crystal to find out the optimal point of harmonic conversion efficiency and realizes the single-point fast measurement and multi-point fast scanning for two kinds of KDP crystals. At the same time, we measured and fitted the linear relationship of detuning angle/peak energy with the sample crystals, and the fitting error was controlled within ±50 urad. We measured the multi-point accuracy, repeatability, and detuning angle of the unknown crystal by MIDC, and compared the measurement results of the unknown crystal with the online adjustment results. The measurement results show that the multi-point accuracy and repeatability of MIDC are within ±20 urad, and its measurement errors for the detuning angle of the unknown crystals have an average value of 72.78 urad, which is 73% higher than that of the previous generation of offline measurement system.

Furthermore, we propose an optimization method for the crystal detuning angle measurement results by studying the facet changes of crystals in online conditions. For the state of crystals in 45° online working condition, we calculate the detuning angle error of KDP crystals under gravity condition using the finite element method.

At the same time, we also investigated the measurement error sources of MIDC. We analyzed the trend of the influence of errors in crystal attitude and optics position on the offline measurement results of the crystals, so as to monitor and adjust for the errors of MIDC.

## Figures and Tables

**Table 1:** Main parameters of components in MIDC.

Item	Value
Laser power	2.2 GW/cm^2^
Focal length of divergent lens	−150 mm
Focal length of convergent lens	200 mm
Measurement range of autocollimator	±7000 μrad
Measurement accuracy of autocollimator	0.1 μrad
Window sizes of CCD	8.8 × 6.6 mm

**Table 2:** Relative position of main components.

Item	Length
Laser to divergent lens	1500 mm
Divergent lens to SHG	165 mm
SHG to THG	50 mm
THG to convergent lens 1/2	270/320 mm
Convergent lens 1/2 to CCD 1/2	435/485 mm

## Data Availability

Data are contained within the article.

## References

[1] MacGowan B.J. The National Ignition Campaign on NIF. Proceedings of the Conference on Lasers and Electro-Optics 2010.

[2] Lindl J.D., Moses E.I. (2011). Special Topic: Plans for the National Ignition Campaign (NIC) on the National Ignition Facility (NIF): On the threshold of initiating ignition experiments. Phys. Plasmas.

[3] Moses E.I. Ignition on the National Ignition Facility. Proceedings of the 5th International Conference on Inertial Fusion Sciences and Applications (IFSA 2007).

[4] Moses E.I., Lindl J.D., Spaeth M.L., Patterson R.W., Sawicki R.H., Atherton L.J., Baisden P.A., Lagin L.J., Larson D.W., MacGowan B.J. (2017). Overview: Development of the National Ignition Facility and the Transition to a User Facility for the Ignition Campaign and High Energy Density Scientific Research. Fusion Sci. Technol..

[5] Moses E.I. (2010). The National Ignition Facility and the National Ignition Campaign. IEEE Trans. Plasma Sci..

[6] Miquel J.L., Lion C., Vivini P. The Laser Mega-Joule: LMJ & PETAL status and Program Overview. Proceedings of the 8th International Conference on Inertial Fusion Sciences and Applications (IFSA).

[7] Deng X.W., Zhu Q.H., Zheng W.G., Wei X.F., Jing F., Hu D.X., Zhou W., Feng B., Wang J.J., Peng Z.T. Research and construction progress of SG-III laser facility. Proceedings of the Conference on High-Power Lasers and Applications VII.

[8] Shiraga H., Fujioka S., Nakai M., Watari T., Nakamura H., Arikawa Y., Hosoda H., Nagai T., Koga M., Kikuchi H. (2012). Integrated experiments of fast ignition targets by Gekko-XII and LFEX lasers. High Energy Density Phys..

[9] Zylstra A.B., Hurricane O.A., Callahan D.A., Kritcher A.L., Ralph J.E., Robey H.F., Ross J.S., Young C.V., Baker K.L., Casey D.T. (2022). Burning plasma achieved in inertial fusion. Nature.

[10] Wegner P., Auerbach J., Biesiada T., Dixit S., Lawson J., Menapace J., Parham T., Swift D., Whitman P., Williams W. NIF final optics system: Frequency conversion and beam conditioning. Proceedings of the 2nd Annual Conference on Optical Engineering at the Lawrence Livermore National Laboratory.

[11] Pritula I., Kosinova A., Kolybayeva A., Puzikov V., Bondarenko S., Tkachenko V., Tsurikov V., Fesenko O. (2008). Optical, structural and microhardness properties of KDP crystals grown from urea-doped solutions. Mater. Res. Bull..

[12] Chen H.F., Dai Y.F., Zheng Z.W., Gao H., Li X.P. (2011). Effect of Crystallographic Orientation on Cutting Forces and Surface Finish in Ductile Cutting of KDP Crystals. Mach. Sci. Technol..

[13] Wang S.F., An C.H., Zhang F.H., Wang J., Lei X.Y., Zhang J.F. (2016). An experimental and theoretical investigation on the brittle ductile transition and cutting force anisotropy in cutting KDP crystal. Int. J. Mach. Tools Manuf..

[14] Barker C.E., Van Wonterghem B.M., Auerbach J.M., Foley R.J., Murray J.R., Campbell J.H., Caird J.A., Speck D.R., Woods B.W. Design and performance of the Beamlet laser third-harmonic frequency converter. Proceedings of the 1st Annual International Conference on Solid-State Lasers for Application to Inertial Confinement Fusion.

[15] Hibbard R.L., Michie R.B., Summers M.D., Liou L.W. Development of a metrology instrument for mapping the crystallographic axis in large optics. Proceedings of the 13th Annual Meeting of the American-Society-for-Precision-Engineering.

[16] Summers M.D., Hibbard R.L., Liou L.W., Mayor W.F., Michie R.B. CAVE: The Design of a Precision Metrology Instrument for Studying Performance of KDP Crystals. Proceedings of the Optical Fabrication and Testing.

[17] Kong C., Ji L., Zhu J. (2010). Design and Precision Analysis of Offline Crystal Frequency Tripling Alignment Adjusting System for SG-Ⅱ. Laser Optoelectron. Prog..

[18] Liu Z., Xu X., Xiong Z., Cao T., Chen N., Ye H., Yuan X., Zheng W., Fan Y., Yi C. (2015). Quick and Accurate KDP Crystal Angle Measuring Method, Involves Determining Accurate Angle of KDP Crystal by Adopting Accurate KDP Crystal Angle Calculating Technique, and Calculating Sum of Relative Transfer Rate of Measuring Point. CN Patent.

[19] Pei G.Q., Du W.F., Ye L., Xu X. (2019). A precision crystal phase-matching angle metrology instrument. Opt. Laser Technol..

[20] Huaiwen G., Wei Z., Lang Y., Weifeng D., Ning T., Xuewei D., Xiaoxia H., Bowang Z., Wei Z., Fang W. (2020). Effect of crystals’ surface shape distortion on conversion efficiency of third harmonic generation. High Power Laser Part. Beams.

[21] Barker C., Auerbach J., Adams C., Bumpas S., Hibbard R., Lee C., Roberts D., Campbell J., Wegner P., Van Wonterghem B. (1997). National Ignition Facility Frequency Converter Development.

